# Neuroendocrine Immunoregulation in Multiple Sclerosis

**DOI:** 10.1155/2013/705232

**Published:** 2013-12-08

**Authors:** Nathalie Deckx, Wai-Ping Lee, Zwi N. Berneman, Nathalie Cools

**Affiliations:** Laboratory of Experimental Hematology, Vaccine and Infectious Disease Institute (Vaxinfectio), Faculty of Medicine and Health Sciences, University of Antwerp, Antwerp University Hospital (UZA), 2650 Edegem, Belgium

## Abstract

Currently, it is generally accepted that multiple sclerosis (MS) is a complex multifactorial disease involving genetic and environmental factors affecting the autoreactive immune responses that lead to damage of myelin. In this respect, intrinsic or extrinsic factors such as emotional, psychological, traumatic, or inflammatory stress as well as a variety of other lifestyle interventions can influence the neuroendocrine system. On its turn, it has been demonstrated that the neuroendocrine system has immunomodulatory potential. Moreover, the neuroendocrine and immune systems communicate bidirectionally via shared receptors and shared messenger molecules, variously called hormones, neurotransmitters, or cytokines. Discrepancies at any level can therefore lead to changes in susceptibility and to severity of several autoimmune and inflammatory diseases. Here we provide an overview of the complex system of crosstalk between the neuroendocrine and immune system as well as reported dysfunctions involved in the pathogenesis of autoimmunity, including MS. Finally, possible strategies to intervene with the neuroendocrine-immune system for MS patient management will be discussed. Ultimately, a better understanding of the interactions between the neuroendocrine system and the immune system can open up new therapeutic approaches for the treatment of MS as well as other autoimmune diseases.

## 1. Introduction

Multiple sclerosis (MS) is a chronic inflammatory autoimmune disease of the central nervous system (CNS). It is characterized by inflammation, demyelination, axonal degeneration, and gliosis. MS affects 1 out of 1000 people in the Western world and leads to chronic disability in mostly young adults (20–40 years). This neurodegenerative disease is characterized by a heterogeneous clinical course with motor sensory and sensible disturbances [1]. The majority of patients (85%–90%) starts with relapses followed by remissions (i.e., relapsing-remitting (RR)-MS). Relapses are a defining feature of MS and reflect focal inflammatory events. With time and age, most patients switch to a progressive phase with gradual deterioration of neurological functions due to progressive axonal degeneration (i.e., secondary progressive (SP)-MS). About 10%–15% of MS patients are diagnosed with primary progressive MS (PP-MS). This progressive form is characterized by a gradual clinical decline in functions with no distinct remissions.

Although MS is considered to be a predominantly immune-mediated demyelinating disease, as demonstrated by immune cell infiltration and accompanying inflammatory processes leading to damage of myelin, the etiology of MS is unknown. It is now generally accepted that MS is a complex multifactorial disease involving genetic and environmental factors affecting the autoreactive immune responses [2]. In this respect, we will address here the role of the neuroendocrine system in MS. Several studies have addressed the possible role of the neuroendocrine system in susceptibility and severity of autoimmune diseases. Moreover, it has been shown that the neuroendocrine system has immune-modulatory potential [3]. Ultimately, a better understanding of the interactions between the neuroendocrine system and the immune system can open up new therapeutic approaches for the treatment of autoimmune diseases, including MS.

## 2. The Neuroendocrine-Immune System

The neuroendocrine system is based on interactions between the nervous and the endocrine system. Furthermore, the neuroendocrine system can both directly and indirectly influence the developmental and functional activity of the immune system. In turn, the immune system can collaborate in the regulation of endocrine activity. The bidirectional interactions between aforementioned systems are known as the neuroendocrine-immune system. The integration between these two systems is essential in order to maintain homeostasis and health. Neuroendocrine regulation of immune responses is important for survival during both physiological and mental stress. Systemically, this regulation is accomplished by hormones, such as those from the hypothalamic-pituitary-adrenal (HPA) axis and the hypothalamic-pituitary-gonadal (HPG) axis. Regional regulation is accomplished by innervations, including the autonomic nervous system, while local regulation is accomplished by neurotransmitters [4]. The immune system regulates the CNS through immune mediators and cytokines that can cross the blood-brain barrier (BBB), or signal indirectly through the vagus nerve or second messengers. Furthermore, an entire constellation of neurotransmitters and neuroendocrine hormones is known to be endogenously produced by the immune system, while the hypothalamus and/or anterior pituitary have been shown to express interleukin (IL)-1, IL-6, transforming growth factor (TGF)-*β*, and other cytokines. Additionally, immune, endocrine, and neural cells express receptors for hormones, cytokines, and neurotransmitters. Hence, these products act in an autocrine, paracrine, and endocrine manner thereby further supporting the postulated bidirectional interactions of the neuroendocrine-immune system [5]. In summary, the neuroendocrine and immune systems communicate bidirectionally via shared receptors and shared messenger molecules, variously called hormones, neurotransmitters, or cytokines.

## 3. Regulation of the Immune System by the Neuroendocrine System and Dysfunction in MS

In a healthy individual, the neuroendocrine and the immune system provide a finely tuned regulatory system. Disturbances of these regulatory systems could potentially lead to oversuppression of the immune system for example, resulting in a higher susceptibility to cancer and infectious diseases, or overactivation of the immune system which on its turn may lead to a higher risk for inflammatory or autoimmune diseases.

### 3.1. The Hypothalamic-Pituitary-Adrenal (HPA) Axis

In order to survive, organisms maintain a complex dynamic equilibrium or homeostasis which is constantly challenged by intrinsic or extrinsic factors such as emotional, psychological, traumatic, or inflammatory stress. For several decades, it has been known that the hormonal stress response is mainly coordinated by the HPA axis. The HPA axis is a regulatory system, including the hypothalamus, pituitary, and adrenal glands and regulatory neural inputs, which functions on both a neuronal and an endocrine level through the release of neural factors and hormones. It has central and peripheral actions, mediates the coordination of circadian events such as the sleep/wake cycle, and helps with coping, adaptation, and recovery from stress.

**Figure 1:**
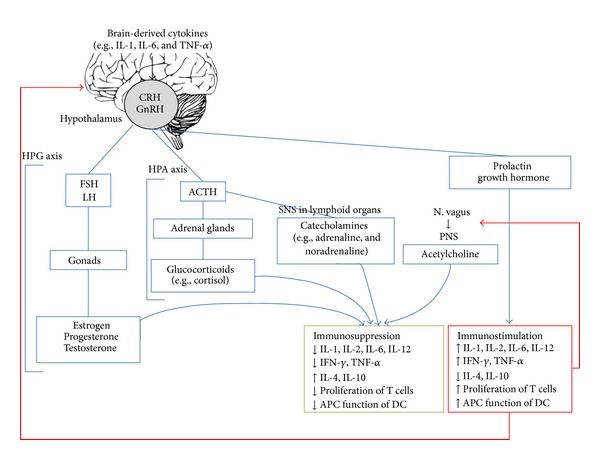
The neuroendocrine-immune system. Via a complex system of common messenger molecules and receptors, the neuroendocrine and the immune systems bidirectionally communicate and monitor each other's activities. Integration of these signals is essential to maintain homeostasis and health and may result in immunosuppression or immunostimulation. Discrepancies at any level can lead to changes in susceptibility to and severity of several autoimmune and inflammatory diseases.

During various physical and psychological stimuli, the HPA axis is activated which results in secretion of corticotrophin-releasing hormone (CRH) and arginine vasopressin (AVP) from the paraventricular nucleus (PVN) of the hypothalamus into the hypophyseal portal blood supply. CRH acts on the anterior pituitary gland to stimulate the release of adrenocorticotropic hormone (ACTH). Subsequently, ACTH circulates through the systemic circulation towards the adrenal cortex where it induces the expression and release of glucocorticoids (GC) in a diurnal pattern (Figure 1). The secretion of CRH is upregulated by serotonergic [6], cholinergic [7], and catecholaminergic systems [8]. On the other hand, opiates and *γ*-aminobutyric acid (GABA) as well as hormones downstream of CRH, such as GC and ACTH, can inhibit the secretion of CRH via negative feedback [9].

It is known that GC, which are amongst the best-characterized hormones, exert a wide variety of immunomodulatory effects, including modulation of cytokine expression, cell adhesion and migration, and production of inflammatory mediators [10, 11]. The immunomodulatory effects of GC are regulated through intracellular glucocorticoid receptors which have a widespread distribution throughout various tissues. There are two different types of glucocorticoid receptors including the high affinity type 1 mineralocorticoid receptor (MR) which mediates non-stress-related circadian fluctuations in GC and is primarily activational. In contrast, the low affinity glucocorticoid receptor (GR) mediates stress levels of GC and is inhibitory in some systems, while being activational in others [12]. Although GC are generally immunosuppressive at pharmacological concentrations, GC are immunomodulatory at physiological levels. Upon ligation, the transcription of target genes is directly and/or indirectly affected by binding of the GR to specific sequences of DNA, known as GC-responsive elements (GRE). In this perspective, GC specifically regulate the immune response causing a shift from T helper type 1 (Th1) to Th2 immune responses. Indeed, GC directly inhibit the production of pro-inflammatory cytokines, such as IL-1, IL-6 and Th1-related cytokines (IL-2, IL-12, and IFN-*γ*) as well as inflammatory mediators, such as prostaglandin and nitric oxide [10], while GC increase the production of anti-inflammatory Th2-related cytokines (IL-4 and IL-10). In doing so, GC enhance immunoglobulin production [13, 14]. Besides, GC have a direct inhibitory effect on the expression of adhesion molecules such as intercellular adhesion molecule-1 (ICAM-1) and E-selectin. These adhesion molecules play a key role in the trafficking of inflammatory cells to sites of inflammation [15]. Furthermore, GC negatively affect dendritic cells (DC), the most specialized antigen-presenting cells (APC), by suppressing their maturation and by downregulating the expression of the major histocompatibility complex (MHC) molecules [16]. On the other hand, GC can indirectly suppress immune responses through the inhibition of pro-inflammatory transcription factors such as nuclear factor kappa-light chain enhancer of activated B cells (NF-*κ*B) [17] and activating protein-1 (AP-1) [18]. NF-*κ*B promotes the expression of the genes coding for many cytokines, enzymes, and adhesion molecules involved in inflammatory diseases [19]. Hence, also the inhibition of the activation of NF-*κ*B contributes to the anti-inflammatory actions of GC.

A well-known GC is cortisol, often referred to as the stress hormone and a powerful natural immunosuppressor. Following binding to glucocorticoid receptors, cortisol is involved in several regulatory functions such as glucose metabolism, regulation of blood pressure, insulin release for blood sugar maintenance, immune function, and inflammatory responses. For example, studies have shown that cortisol can prevent T cell proliferation by downregulation of the IL-2 receptor [20]. During the body's fight or flight response to stress, cortisol is secreted at higher levels and is responsible for several stress-related changes in the body. Moreover, this immunosuppressive hormone plays an important role in the circadian rhythm as its plasma levels exhibit a diurnal pattern with peak levels in the morning at approximately 9 am and a nadir at night [21]. Interestingly, some cytokine concentrations also follow a diurnal rhythm. Proinflammatory mediators in serum, such as IL-1, IL-6, and soluble IL-2 receptors, peak at 1–4 am and are low throughout the day with a nadir at 8–10 am when cortisol levels are the highest [22–24]. Interestingly, circadian involvement has been noted in various autoimmune and inflammatory diseases [25]. Indeed, Cutolo et al. have documented that clinical signs and symptoms of patients with rheumatoid arthritis (RA) vary within a day [25]. More severe symptoms are often presented upon waking in the morning possibly associated with peak levels of pro-inflammatory cytokines during the night. Melatonin, which antagonizes the immunosuppressive effects of cortisol, is secreted by the pineal gland in the brain. Melatonin levels begin to rise in the midevening to late evening, peak at approximately 3 am, and then drop in the early morning hours. It has been demonstrated that melatonin production in RA patients is increased in comparison with healthy controls at the beginning of the night and in the early morning and is correlated with the typical peak of joint stiffness and pain.

Clinical and experimental studies have demonstrated that abnormalities in the HPA axis in MS may contribute to enhanced susceptibility to disease and to more severe disease activity [26–28]. Although experimental data in experimental autoimmune encephalomyelitis (EAE), the most commonly used animal model of MS, have suggested low reactivity of the HPA axis as a predisposing factor for disease susceptibility and severity [29, 30], it has been demonstrated that up to 50% of MS patients are endowed with HPA axis hyperactivity [31]. Basal plasma levels of cortisol and ACTH were found to be elevated [32] and adrenal glands were demonstrated to be enlarged in MS patients [33]. It was shown that after CRH stimulation, the cortisol response varied according to the disease status of the MS patient and was lower in SP-MS patients compared to patients with PP-MS and healthy controls, while a higher *β*-endorphin/ACTH response was found in RR-MS patients as compared to other groups [34]. Moreover, higher cortisol levels were often determined during or in close proximity to acute relapse, which is characterized by an MRI-confirmed inflammatory state [26, 27, 34, 35] and correlated with higher white blood cell counts in the cerebrospinal fluid (CSF) [26]. In addition, histopathological findings of the hypothalamus reveal perturbations in CRH regulation as a result of MS lesions in this area. Indeed, an elevated number and activity of CRH-immunoreactive neurons co-expressing vasopressin (i.e., CRH/VP neurons) were found in the hypothalamus of MS patients compared to controls in postmortem studies [33, 36, 37]. Whereas these observations were confirmed by Huitinga and colleagues, they additionally reported an inverse correlation between active MS lesions and the number of hyperactive CRH/VP neurons and levels of CRH mRNA [28]. Furthermore, they observed a more severe disease course in MS patients with high active lesions in the hypothalamus and the lowest CRH expression, suggesting impaired cortisol secretion and reduced ability to control inflammation. The authors hypothesized that this effect was mediated by APC present in the active lesion suppressing the CRH/VP neurons thereby contributing to a more severe disease. Noteworthy, CRH mRNA levels return to normal during remission [29].

Altogether, the HPA axis hyperactivity in MS has been accompanied with progressive disease and global neurodegeneration [38]. Experimental studies suggest that stress and excessive levels of GC may contribute to cellular and molecular disturbances in the brain which may lead to damage in several brain areas including the hippocampus. Indeed, Gold et al. observed smaller hippocampal volumes in MS patients as compared to healthy controls [39]. Given the important role of the hippocampus in learning, mood regulations, memory, and the HPA axis control, as well as the notion that fatigue and depression are among the most common symptoms of MS, significant associations between HPA axis activity and depressive symptoms have been observed in RR-MS during relapse [26]. Gold et al. have detected normal morning but increased evening cortisol levels in MS patients with depressive symptoms compared to non-depressed MS patients [40] as well as compared to age- and gender-matched healthy controls [39]. Although overall RR-MS patients expressed a significantly higher cortisol awakening response compared to healthy controls, only RR-MS patients with moderately elevated depression scores showed significant differences in their cortisol release, while RR-MS patients with low depression scores expressed similar circadian patterns as healthy controls [41].

Besides the release of GC including cortisol, the HPA axis also regulates the secretion of prolactin and growth hormone (GH). Accordingly, these hormones exhibit immunoregulatory effects. Briefly, through stimulation by suckling and stress, prolactin is released from the anterior pituitary gland and stimulates mammary growth and differentiation. Moreover, it is documented that prolactin has immunostimulatory effects such as increasing the production of IFN-*γ* and IL-12 and the proliferation of T cells [42, 43]. On the other hand, GH mediates its effect through insulin-like growth factor-1 (IGF-1) [44]. Both GH and IGF-1 modulate the immune system by inducing the survival and proliferation of lymphoid cells [45]. In addition to these well-described effects on adaptive immunity, prolactin and GH also modulate innate immunity. Indeed, both hormones enhance activation of macrophages and induce subsequent release of reactive oxygen species (ROS) [46, 47].

### 3.2. The Hypothalamic-Pituitary-Gonadal (HPG) Axis

In addition to the HPA axis, other central hormonal systems, such as the HPG axis, modulate the immune system [48]. To date, it is generally accepted that gender affects the susceptibility and course of autoimmune diseases. Whereas almost 8% of the world population develops an autoimmune disease, approximately 78% of them are women. Also, MS has a higher prevalence, but better prognosis in women than in men [49].

The integrating center of this reproductive hormonal axis is the hypothalamus. Gonadotropin-releasing hormone (GnRH) is synthesized and released by the hypothalamus into the hypophyseal-portal circulation. Upon transport to the pituitary gland, GnRH stimulates the synthesis and secretion of gonadotropic hormones including follicle-stimulating hormone (FSH) and luteinizing hormone (LH) which following systemic release circulate towards the reproductive organs and subsequently stimulate the release of estrogen and progesterone.

Estrogen is a potent steroid with pleiotropic effects and is present in high levels in females from adolescence to menopause. There are 3 naturally occurring estrogens: estrone (E1), estradiol (E2), and estriol (E3) which are the predominant forms during menopause, in non-pregnant females, and during late pregnancy, respectively. Estriol has been accepted as the safest of the three and has been used worldwide for the treatment of menopausal symptoms [50, 51]. Estrogen exerts its effect through binding to two forms of nuclear estrogen receptors (ER), ER*α* and ER*β*, which exhibit distinct transcriptional properties. ER*α* is expressed on the endometrium, ovarian stromal cells, breast, and hypothalamus, whereas ER*β* is widely expressed in tissues including brain, kidney, bone, heart, lungs, intestine, and endothelial cells [52]. In addition, expression of ER has been demonstrated in a variety of immune cells including monocytes, neutrophils, DC, T cells, and B cells, thereby providing indirect evidence for its immunomodulatory properties [53, 54]. Following ligation, the ER interacts with the transcription factor NF-*κ*B, thereby affecting secretion of cytokines, chemokines, and matrix metalloproteinase (MMP)-9, as well as antigen presentation and function of DC [55]. More specifically, pretreatment with 17*β*-estradiol blocked the ability of DC to present antigen to T cells resulting in an altered pattern of cytokine production, as evidenced by an increase of Th2 cytokines, such as IL-10 and IL-4, and a decrease of Th1 cytokines including TNF-*α* and IFN-*γ*. Furthermore, it was shown that 17*β*-estradiol treatment significantly decreased the frequency of DC migrating towards the CNS at the onset of EAE [56, 57]. Likewise, estriol decreased the secretion of MMP-9 by immune cells thereby abrogating subsequent migration of inflammatory cells towards the CNS [58]. This effect may be indirectly mediated through downregulation of TNF-*α* [59], which activates MMP-9 [60]. In addition to their anti-inflammatory effects, estrogens also appear to be neuroprotective in several CNS disorders such as MS, Alzheimer's disease, Parkinson's disease, and spinal cord injury [61–64], as evidenced by improvement of clinical disease and reduction of neuropathology following estrogen treatment. Reported neuroprotective effects are inhibition of neuronal loss by decreasing glutamate-induced apoptosis [65] and protection of oligodendrocytes from cytotoxicity [66] as well as stimulation of oligodendrocyte function [67] and upregulation of TGF-*β* production from astrocytes [68].

Furthermore, pregnancy, postpartum period, and menopause as well as other physiological conditions have been demonstrated to affect the clinical course of a variety of autoimmune disorders. These clinical observations suggest the importance of sex hormones in immune modulation. Several studies have documented that, during pregnancy, both clinical symptoms and relapse rate of MS are decreased, whereas the postpartum period is associated with a higher risk for exacerbation of the disease [69, 70]. This suggests a role for the hormones fluctuating at this time, such as, cortisol, progesterone, and estrogen, in the regulation of MS activity [71]. It may be clear that pregnancy induces changes in the maternal immune system in order to protect the foetus. The increase of estrogen, progesterone, and cortisol, during pregnancy is associated with increased production of Th2 cytokines and decreased production of Th1 cytokines. Hence, the improvement of MS symptoms during pregnancy may be linked to a shift from the prevailing Th1 response to a Th2 response, while postpartum worsening may be associated with the return to the Th1 environment [72]. The improvement of symptoms occurs predominantly during the third trimester of pregnancy when circulating estrogen and progesterone levels peak, while the postpartum period is characterized by an abrupt drop in estrogen levels. It needs to be noted that, consistent with these findings, hyperestrogenic states are associated with disease flareup of systemic lupus erythematosus (SLE) in which Th2-mediated humoral response is an important pathogenic factor [73].

Sex differences have also been observed in EAE. Female mice are more susceptible to EAE than males, albeit that a genetic background may also influence the effects of sex hormones on the immune system [74]. Interestingly, the minimal effective estrogen dose that inhibits EAE varies greatly between mouse strains [75] suggesting that estrogen receptor sensitivity may influence MS risk. In addition, it has been reported that ER*α* ligand treatment can ameliorate EAE by decreasing pro-inflammatory cytokines, such as TNF-*α* and IFN-*γ*, while enhancing the secretion of the anti-inflammatory cytokine IL-5. Furthermore, reduced CNS white matter inflammation, protection against axonal loss, and demyelination in EAE were documented [76].

In summary, the numerous immunomodulatory and neuroprotective effects of estrogens can attribute to their protection in several neurodegenerative and autoimmune diseases. Next to estrogens, other hormones released through the HPG axis exert immunoregulatory effects. Briefly, high levels of prolactin have been described in MS patients [77], resulting in increased production of IFN-*γ* and IL-2 by Th1 cells and autoantibody production through activation of Th2 cells [78]. In addition, testosterone inhibits both innate and adaptive immunity. It has been reported that testosterone can enhance production of IL-5 and IL-10 and decrease IFN-*γ* production by T cells *in vitro* thereby promoting a Th2 response [79]. Treatment with androgen significantly delayed onset and progression of EAE [80–82]. The protective effects of androgens were accompanied with decreased production of Th1 cytokines [82] and increased production of anti-inflammatory IL-10 [80] as well as inhibition of T cell infiltration into the spinal cord [83]. The protective effects of androgens were further confirmed by the observation that, in human male MS patients, low testosterone levels but higher estradiol levels are associated with a higher degree of brain tissue damage [84]. On the other hand, progesterone exerts anti-inflammatory effects by inhibiting NF-*κ*B and enhancing IL-4 production [85, 86]. In EAE, progesterone treatment results in a decreased production of inflammatory IL-2 and IL-17 and an increased production of IL-10 resulting in attenuated disease severity [87]. Furthermore, progesterone enhanced axonal density and reduced axonal damage in EAE [88].

### 3.3. Neuronal Pathways

#### 3.3.1. Regional Regulation by Innervations

Regional regulation of the immune system through the autonomic nervous system is mediated by innervations of primary and secondary lymphoid organs. Furthermore, T cells, B cells, and DC are located adjacent to nerve terminals. Depending on the pathological conditions, innervation of lymphoid organs can change. For example, the number of innervations in lymphoid organs increases under psychosocial stress in primates, whereas it decreases following viral infection [89].

(1) *Sympathetic Nervous System.* The catecholamines, adrenalin and noradrenalin, are released from sympathetic nerve terminals upon stimulation. Stress situations, such as a physical threat, excitement, a loud noise, or a bright light, are the major physiological triggers of the release of catecholamines. These stimuli are processed by the CNS through release of ACTH. Subsequently, ACTH stimulates the synthesis of adrenalin and noradrenalin both directly as well as indirectly via cortisol production. Through the release of catecholamines in lymphoid organs, the sympathetic nervous system (SNS) has been demonstrated to exert a direct role in immunomodulation. Whereas most studies have demonstrated that activation of the SNS inhibits the immune system, some studies show opposite effects including induction of chemokines [90]. This possible paradigm can be explained by various actions of adrenalin and noradrenalin through ligation of different receptors. Indeed, the stimulation of *α*-adrenoreceptors (*α*1AR) is predominantly associated with immunostimulatory effects on immune cells, for example, IL-1*β* secretion by human monocytes and macrophages [91], which attributes to many chronic inflammatory disease states [92]. In contrast, stimulation of *β*-adrenoreceptors has suppressive actions. Stimulation of *β*2-adrenergic receptors (*β*2AR) on DC and macrophages upregulates cyclic AMP (cAMP), activates protein kinase A, and inhibits the transcription factor NF-*κ*B, thereby affecting cytokine production. For example, production of pro-inflammatory cytokines, such as TNF-*α*, IL-1, IL-6, and IL-12, is downregulated, while production of the anti-inflammatory cytokine IL-10 is upregulated [93, 94]. These events result in the suppression of Th1 responses. In addition, adrenalin and noradrenalin influence other innate immune cells, such as NK cells, by reducing NK cell activity directly as well as indirectly through the inhibition of IL-12 and IFN-*γ*. Moreover, adrenalin and noradrenalin suppress the migration, phagocytosis, and degranulation of neutrophils [95].

Several studies have indicated the involvement of catecholamines in the pathogenesis of MS, as demonstrated by increased *β*-adrenergic receptor density on peripheral blood mononuclear cells (PBMC) from RR-MS patients [96] and discrepant noradrenalin and adrenalin levels in the PBMC from MS patients [97]. In addition, experimental studies in EAE have shown that selective depletion of noradrenalin levels in the CNS resulted in exacerbated clinical scores. Selective increase of CNS noradrenalin levels reduced astrocyte activation in the molecular layer of the cerebellum without affecting splenic Th1 or Th17 immune responses, thereby possibly providing benefit in EAE without affecting peripheral immunity.

Dopamine, another catecholaminergic neurotransmitter, also has important functions in the peripheral nervous system, as indicated by its release from peripheral nerve endings innervating lymphoid organs as well as from immune cells. Dopamine receptors are classified into two subgroups, dopamine-1 (D1)-like receptors (D1R and D5R) and D2-like receptors (D2R, D3R and D4R) [98]. In general, D1- and D2-like receptors are coupled to stimulation and inhibition of intracellular cAMP production, respectively [99]. In doing so, D1-like receptor-mediated increase of intracellular cAMP impairs the function of cytotoxic T lymphocytes (CTL) and regulatory T cells (Treg) [100, 101]. In contrast, it was reported that stimulation of D1-like receptors is involved in the polarization of naïve CD4^+^ T cells towards Th17 cells [102]. D2-like receptor-mediated modulation of T cell function is demonstrated by IL-10-dependent induction of Treg [103], secretion of TNF-*α* from T cells indicative of a Th1 effector phenotype [103], and the differentiation of naïve CD8^+^ T cells into CTL [103] as well as the modulation of the homing of T cells [104].

Similar to noradrenalin, dopamine levels are decreased in autoimmunity [105], suggestive of a protective role in the regulation of MS. Indeed, administration of a D2-like receptor agonist attenuates both the acute and the late phase of EAE [106], while administration of D2-like receptor antagonists worsened EAE pathology [102]. On the other hand, administration of D1-like receptor antagonists ameliorated EAE, which was associated with reduced IL-17 and increased IFN-*γ* levels. This finding was supported by previous results suggesting that dopamine signaling via D1-like receptors aggravates Th17-mediated diseases, such as MS, by promoting the IL-6/Th17 axis in conjunction with the suppression of Treg. Altogether, it is likely that D1-like receptors expressed on T cells are involved in the interface between autoimmunity and health. Indeed, decreased levels of D5R mRNA and protein have been found in PBMC from MS patients as compared to controls [107]. Noteworthy, dopamine reduced MMP-9 mRNA in controls and in IFN-*β*-treated MS patients, but not in untreated MS patients [107].

(2) *Parasympathetic Nervous System.* Acetylcholine (ACh) is the primary neurotransmitter of the parasympathetic nervous system (PNS). The PNS modulates immune responses through the efferent and afferent fibers of the vagus nerve. Two mechanisms demonstrating the inhibitory activity of the PNS on innate immune cells have been described [108].

First, direct stimulation of paraganglia cells by inflammatory cytokines, such as IL-1, results in signaling through afferent fibers. This leads to activation of parasympathetic brainstem regions to release ACh from efferent vagus nerves, thereby controlling inflammation through negative feedback. Subsequent binding of ACh to nicotinic receptors blocks the NF-*κ*B signaling pathway. For example, stimulation of the *α*7-nAChR on macrophages, lymphocytes, and neutrophils inhibits NF-*κ*B transcriptional activity and the production of inflammatory cytokines [109]. In addition, *α*4*β*2-nAChR activation modulates endocytosis and phagocytosis by macrophages [110]. Alternatively, ACh binds to the muscarinic ACh receptors (mAChaR). The M3 mAChR is expressed on T cells and has a role in the regulation of adaptive immune responses. Upon T cell receptor (TCR) stimulation, T cells release ACh, which stimulates M3 mAChR in an autocrine manner, thereby potentiating T cell activation and favoring differentiation towards a Th1 phenotype [111]. Hence, it can be summarized that the immunosuppressive or immunostimulatory consequences of ACh are dependent on the receptor type involved.

The second mechanism is indirect. When the peripheral cytokine-mediated inflammatory reaction stimulates the afferent sensory vagal route, a reflex response through the HPA axis that releases ACTH and GC is activated, which in turn reduces the production of pro-inflammatory cytokines.

A major region of cholinergic input, which plays an important role in learning and memory function, consists in the basal forebrain in the hippocampus [112]. Since the hippocampus is severely affected in MS patients as aforementioned [39], a selective imbalance in the hippocampal cholinergic neurotransmission exists in MS patients [113]. Accordingly, reduced synthesis of ACh is observed, possibly contributing to memory complaints as experienced by a significant proportion of MS patients [114].

#### 3.3.2. Local Regulation by Neurotransmitters

Local regulation of the immune system is mediated by neurotransmitters which are synthesized in neurons and act on the postsynaptic neurons and other organs. Neurotransmitters are released from both the CNS and the peripheral nervous system as well as from immune cells including T cells, B cells, macrophages, DC, and granulocytes [4] thereby underscoring their possible contribution to the modulation of immune responses.

(1) *Glutamate.* Glutamate is a primary excitatory neurotransmitter in the CNS and has direct impact on neuronal activity [115]. Glutamate binds to ionotropic glutamate receptors (iGluR) or to metabotropic glutamate receptors (mGluR). Some G protein-coupled mGluR were recently reported to be involved in immune responses. For example, the expression of mGlu1R is induced after T cell activation and its ligation enhances the secretion of IL-2, IL-6, IL-10, TNF-*α*, and IFN-*γ*. In contrast, stimulation of mGlu5R, which is constitutively expressed on T cells, inhibits T cell proliferation through suppression of IL-6 production [116]. Hence, mGlu1R signaling counteracts the mGlu5R-mediated inhibitory effect on T cell proliferation.

Recent studies have identified glutamate as an important determinant of neurodegenerative damage in the course of MS [117]. It was shown that MS patients have increased glutamate levels in the brain [118] and in the CSF [119]. Furthermore, expression of iGluR and transporters is disturbed in MS [120] and in EAE [121]. Loss of glutamate transporters in cortical lesions correlates with microglial activation and synaptic damage [122]. In addition, overactivation of iGluR causes MS-like lesions [123], whereas iGluR antagonists exert beneficial effects in MS [124] and EAE by limiting oligodendrocyte and neuronal damage [125]. This increase in glutaminergic transmission observed in MS patients leads to excitotoxicity and neurodegeneration, resulting in cognitive impairments during the early phase of MS pathogenesis before the appearance of severe motor impairments. However, these actions may also be a consequence of a simultaneous dysfunction of GABA transmission, causing an imbalance between synaptic excitation and inhibition. Indeed, increased glutamate-mediated transmission and loss of GABAergic inputs were observed in EAE [126].

Besides, mGluR are also likely to contribute to glutamate transmission changes in MS and EAE. Indeed, it has been reported that mGlu1R expression in the cerebellum of MS patients and of mice with EAE is lower in comparison with controls, while the expression of mGlu5R is increased [127]. However, active MS lesions are characterized by increased expression of both receptors as well as the expression of mGlu2, -3, -4, and -8 [120]. Paradoxically, experimental studies in EAE have shown protective effects of these receptors. Indeed, treatment with a mGlu1R-selective enhancer resulted in ameliorated motor performance in EAE [127]. In addition, mGlu4R-deficient mice were more prone to develop EAE, which was associated with higher Th1/Th17 responses and increased production of inflammatory cytokines, such as IL-6, IL-12, and IL-23 [127]. Moreover, administration of a mGlu4R-selective enhancer increased resistance to EAE by inducing Treg, supporting the immunosuppressive effect of mGlu4R-mediated signaling [128].

(2) *Tachykinins.* Substance P and neurokinin A are closely related neurotransmitters and are both encoded by the same *Tac1* gene. Substance P is produced by the CNS and the peripheral nervous system, as well as by immune cells including monocytes, DC, and lymphocytes. It is a pro-inflammatory modulator of the immune response acting in either autocrine or paracrine fashion via the neurokinin (NK)-1 receptor, which is the primary receptor for substance P. Via activation of NF-*κ*B in monocytes, substance P mediates increased production of pro-inflammatory mediators, such as IL-1*β*, IL-6, TNF-*α*, macrophage inflammatory protein (MIP)-1*β*, and IFN-*γ* [129]. In doing so, T cell proliferation as well as the generation of Th1 and Th17 cells is induced [130]. It was also shown that substance P regulates antigen presentation of DC [131], increases NK cell activity, and induces the release of CXCL8 and CCL2 from leukocytes as well as of vasoactive mediators, such as serotonin and histamine, from mast cells [132]. In MS plaques, substance P production has been demonstrated in activated macrophages [133] and astrocytes [134]. Although this may indicate a possible role for substance P in MS, no difference in substance P levels in the CSF from MS patients could be demonstrated as compared to healthy controls [135]. Whereas substance P directly acts on endothelial cells, resulting in increased vascular permeability [134] and subsequent enhanced permeability of the BBB, no interference with the induction of EAE in* NK-1* ^−/−^ mice could be observed [136]. Conversely, less severe clinical symptoms and reduced inflammation in the receptor-deficient mice were apparent which may indicate that substance P contributes to the maintenance of CNS inflammation during the chronic phase of EAE [136].

The NK-2 receptor exhibits the highest affinity for neurokinin A. Neurokinin A is known to control various vital responses in humans, such as airway contraction, vasodilatation, and vascular permeability [137]. The function of neurokinin A in the immune system is less well defined compared with the role of substance P. One study reported that neurokinin A stimulation induced mRNA expression of type I interferons, upregulated expression of MHC class II molecules, and antigen presentation by DC, thereby enhancing DC function [138] and subsequently inducing CD4^+^ and CD8^+^ T cell responses. Although this suggests involvement of NK-2 receptor-mediated signaling in chronic inflammation by excessive Th1-mediated immunity [138], no data describing a contributing factor of neurokinin A to the development or sustainment of MS have been reported. 

(3) *Serotonin.* The neurotransmitter serotonin, also known as 5-hydroxytryptamine (5-HT), is produced by the CNS and regulates cognitive and endocrine functions, stress reactivity, circadian rhythm, and sleep [139]. Outside the CNS, serotonin is present in platelets, lymphocytes, monocytes, macrophages, mast cells, pulmonary neuroendocrine cells, enterochromaffin cells of the gut, and in some other cell types. Currently, at least 14 genetically, pharmacologically, and functionally distinct serotonin receptors have been identified. Among these, the serotonin-1A and serotonin-2A subtypes are of particular interest since they play a crucial role in the regulation of serotonergic neurotransmission and emotional and behavioral processes as well as the pathophysiology of various neuropsychiatric disorders [140]. These receptors are also expressed on immune cells and receptor activation appears to be both immunostimulatory and suppressive [141]. For example, through binding of the serotonin-1A receptor on monocytes, serotonin abrogates the monocyte-mediated suppression of NK cell functions [142], such as NK cell cytotoxicity, IFN-*γ* production by NK cells, NK cell proliferation, and expression of the CD16/56 NK cell antigen [142]. In contrast, serotonin decreases cAMP levels via the serotonin-1A receptor, which leads to stimulation  of T cell proliferation [143], while ligation of the serotonin-2A receptor resulted in reduced lymphocyte proliferation [144] as well as decreased numbers of CTL [145].

Initial evidence for involvement of serotonin in autoimmunity comes from the experimental autoimmune neuritis (EAN) model. It was shown that blockade of the serotonin transporter by a selective serotonin reuptake inhibitor, thereby increasing the extracellular levels of serotonin, suppressed EAN [146]. Similarly, blockade of serotonin receptors also suppressed the development of EAE [147]. Furthermore, mice deficient for the serotonin transporter showed a milder disease course of EAE as compared to wild-type controls [148]. This was possibly mediated by a serotonin-dependent reduction of the inflammatory infiltrate in the CNS and by a reduction of the neuroantigen-specific production of IFN-*γ* by splenocytes. In addition, during the early paralytic stages of EAE, damage to the bulbospinal serotonergic neurons occurs, whereas neurologic recovery is associated with reestablishment of spinal serotonergic transmission. Damage to the bulbospinal serotonergic fibers also occurs in MS patients. This is reflected by reduced levels of 5-hydroxyindoleacetic acid (5-HIAA), a metabolite of serotonin, in the CSF. Therefore, it is conceivable that degeneration of bulbospinal serotonin axons contributes to various neurologic manifestations of MS including autonomic and sensory symptoms [149].

(4) *Histamine.* Histamine is produced by histaminergic neurons located in the hypothalamus or released by mast cells, basophils, platelets, and enterochromaffin-like cells. Its major effects are related to sleeping, locomotor activity, exploratory behavior, food intake, awakening, and aggressive behavior [150]. Histamine can either inhibit or stimulate inflammatory reactions, depending on the type of receptor stimulated [151].

Upon histamine 1 receptor (H1R) ligation, histamine induces an increment of the secretion of the pro-inflammatory cytokines IL-1*β*, IL-6, and IL-8 and the chemokine CCL5 by peripheral macrophages [152]. Similarly, stimulation of H4R expressed on hematopoietic and immunocompetent cells involved in inflammatory responses also results in increased secretion of pro-inflammatory cytokines [153]. In addition, *in vitro* experiments indicated that histamine promotes Th1 responses through H1R and downregulates both Th1 and Th2 responses through H2R [154]. H1R and H4R ligation on CD4^+^ T cells induces chemotaxis *in vitro*, whereas H1R and H2R modulate cytokine production. Another study indicated that binding of histamine to the H2R expressed on monocytes reduced the release of the pro-inflammatory cytokines IL-12 and TNF-*α*, while production of the anti-inflammatory cytokine IL-10 and Th2-like activity was increased [155]. Interestingly, expression of different histamine receptors is differentially regulated, depending on the stage of differentiation and of activation of target cells thereby potentially explaining variation in experimental data from diverse studies [156].

Already in 1983, it was noted that histamine may be involved in MS, as evidenced by 60% higher histamine levels observed in MS patients as compared to healthy controls [157]. Since then, several experimental studies confirmed the role of histamine in MS. Upregulated expression of H1R was shown in MS lesions [158], whereas epidemiological studies demonstrated a protective effect of H1R antagonists capable to cross the BBB in MS [159]. Further evidence was provided by a study showing the requirement for *Hrh1 *gene expression for susceptibility to EAE [160]. Indeed, H1R-deficient mice exhibit a significant delay in the onset of EAE and a reduction in the severity of clinical signs compared with wild-type mice [160]. In addition to H1R, H2R also seems to partially regulate encephalitogenic Th1 responses and EAE susceptibility. Indeed, H2R^−/−^ mice develop less severe disease than wild-type mice during the acute and early phase [161], possibly mediated by H2R-dependent abrogation of pro-inflammatory cytokine production.

Although H1R and H2R have a clear pro-inflammatory role and disease-promoting effect, H1R and H2R activation may also play an important role in limiting autoimmune responses [162]. It was shown that histamine ligation of H1R and H2R inhibits the proliferation of murine CD3^+^ T cells directed against myelin-derived antigens *in vitro*, as well as their adhesiveness to the inflamed endothelium [163]. Accordingly, treatment with an H2R agonist reduces the clinical signs in EAE [164]. Furthermore, H4R^−/−^ mice develop more severe EAE, accompanied by increased neuroinflammatory signs and increased BBB permeability, with a higher proportion of infiltrating Th17 cells than Treg, as compared to wild-type mice [165].

(5) *Gamma-Aminobutyric Acid.γ*-Aminobutyric acid (GABA) is the most prominent inhibitory neurotransmitter in the CNS [166]. In the immune system, GABA receptors are expressed on lymphocytes [167] and peripheral macrophages [168]. GABA has similar anti-inflammatory actions as GC. Indeed, GABA negatively modulates the levels of pro-inflammatory cytokines produced by macrophages [169] as well as cell proliferation [170] and migration [171].

Loss of GABAergic innervations is a physiologic hallmark of MS and EAE. Additionally, it was shown that GABA is decreased in the serum and CSF of MS patients and in EAE [172, 173]. Reduced GABA-related gene transcripts and density of inhibitory interneuron processes in motor cortex samples from MS patients were also reported [174] as well as irreversible alterations of GABA transmission in the striatum of EAE mice. Increasing GABA concentration in the CNS delayed EAE onset and reduced severity of symptoms following EAE induction. In mice with established EAE, it reversed paralysis and decreased the number of relapses [175]. Moreover, the chronic persistence of pro-inflammatory cytokines in EAE induced profound alterations in the electrophysiological network properties in cultured cortical neurons, which were reverted by GABA administration [176]. This was further supported by demonstrating inhibition of GABA transmission in mouse brain slices upon administration of CSF from MS patients with MRI-confirmed active brain lesions. The investigators concluded that focal inflammation in MS perturbs the cytokine milieu within the CSF, resulting in diffuse GABAergic alteration in neurons [177].

## 4. Regulation of the Neuroendocrine System by the Immune System and Dysfunction in MS

Given the bidirectional interactions of the neuroendocrine and the immune systems, the immune system also regulates the neuroendocrine system through the secretion of cytokines. Cytokines are immune mediators produced in response to antigens and toxins or after stimulation by other cytokines. Cytokines and their receptors are expressed in the neuroendocrine system and exert their effects both centrally and peripherally [178, 179]. Inflammation in the CNS contributes to the onset and progress of neurodegenerative diseases, including MS [180]. Indeed, pro-inflammatory cytokines, such as IL-1, IL-6, and TNF-*α* play an important role during the pathophysiological processes involved in the disease pathogenesis and course of MS. Through several mechanisms, including humoral, neural, and cellular pathways, cytokines are able to reach the brain. On the one hand, they can enter the brain through the areas with a poorly developed BBB or via active transport across the BBB. On the other hand, these cytokines can be expressed and released from resident cells in the CNS, including glial cells, neurons, endothelial cells, or invading immune cells [181]. Moreover, cytokines that are produced in the periphery activate primary afferent nerves, such as the vagus nerves. In doing so, cytokines stimulate neurons to modulate the social interaction [182], the stressful HPA axis responses [183], and the activities of the autonomic nervous system [184].

Excessive pro-inflammatory cytokine production is physiologically joined to a simultaneous increment of the synthesis of anti-inflammatory cytokines, inhibitory neurotransmitters, and GC. The resulting equilibrium is called homeostasis. However, prolonged increased HPA axis activity results in a prompt loss of the anti-inflammatory mediators with an increase of pro-inflammatory mediators [185], thereby ultimately contributing to a state of disease. An altered cytokine balance has been observed in MS patients, as evidenced by increased pro-inflammatory cytokine levels in the periphery and in the CNS. Indeed, elevated mRNA and protein levels of IL-1*β*, IL-6, and TNF-*α* have been reported in CNS lesions, CSF, and peripheral blood monocytes of MS patients [186, 187] as well as in EAE [188]. Additionally, activated astrocytes and microglial cells express a large number of cytokines and chemokines which subsequently contribute to neuroinflammation in MS. These brain-derived cytokines also act to protect from or enhance neuronal cell death. In doing so, cytokine-mediated neuronal cell death is considered to be important in several neurodegenerative diseases, such as MS.

### 4.1. Cytokine-Mediated Regulation of Hormones

Whereas interferons were the first cytokines shown to exert neuroendocrine effects as demonstrated by increased steroid production upon interferon treatment, it is now clear that several cytokines have functions in the neuroendocrine system. Indeed, IL-1, IL-2, IL-6, IL-10, IFN-*β*, IFN-*γ*, leukaemia inhibitory factor (LIF), TNF-*α*, and granulocyte-macrophage colony-stimulating factor (GM-CSF) can stimulate the HPA axis to release GC. In particular, these cytokines have been reported to elevate plasma GC levels in both humans and animal models [189–193] via stimulation of CRH and ACTH production in hypothalamic and pituitary tissues, respectively. In addition, melatonin release by the pineal gland is stimulated by IFN-*γ*, granulocyte colony-stimulating factor (G-CSF), and GM-CSF [194, 195]. In contrast to the HPA axis, inflammatory cytokines have negative effects on the HPG axis, resulting in reduced gonadal functions [196].

### 4.2. Cytokine-Mediated Regulation of Neurotransmitters

Activation of innate immune responses, by pathogens as well as by damage-associated molecules, leads to the release of inflammatory cytokines that signal the CNS via the subdiaphragmatic vagus nerve, thereby resulting in changes that are associated with sickness behavior, such as fever [197]. Pro-inflammatory cytokines, such as IL-1, IL-2, IL-6, IFN-*β*, IFN-*γ*, LIF, and TNF-*α*, stimulate the SNS to release noradrenalin. Furthermore, IL-1*β* enhances the inhibitory effects of GABA. Given the inhibitory effect of these neurotransmitters on inflammation, this negative feedback loop will stop inflammation. In addition, IL-1*β* administered systemically or in the brain resulted in subsequent increased extracellular levels of serotonin in the anterior hypothalamus and in the hippocampus [198].

Several cytokines are also involved in the regulation of sleep and wakefulness [199], including IL-1*β*, IL-1 receptor antagonist, IL-2, IL-2 receptor, IL-4, IL-6, IL-9, IL-10, IL-13, IL-18, TGF-*β*, IFN-*α*, IFN-*γ*, TNF-*α*, and TNF-*α* receptors p55 and p75 [200–203]. Pro-inflammatory cytokines are more likely to induce sleep, whereas anti-inflammatory cytokines show antisomnogenic effects or do not influence sleep-wake regulation.

Chemokines, a large group of proteins from the cytokine family that are pivotal in leukocyte migration, were found to play a role in signaling functions in the CNS [204]. Macrophages, glial cells, and also neurons are able to constitutively express chemokines and multiple chemokine receptors, which may function as neuromodulators in the homeostatic brain. In neurons, chemokines are located in central nerve endings in small clear and dense core vesicles [205], where they colocalize with traditional neurotransmitters and are released following membrane depolarization [206]. CXCL12 and its receptor CXCR4 [207, 208] as well as CCL2 and its receptor CCR2 are constitutively expressed by mesencephalic dopaminergic neurons [209]. Therefore, both chemokines can modulate the electrical activity of dopaminergic neurons. Furthermore, CCL2 can be upregulated by cells surrounding the sites of brain injury and can attract progenitor cells for healing purposes [210].

The aforementioned hormones, neurotransmitters, and cytokines with their immunomodulatory activity are summarized in Table 1. A comprehensive overview of the interaction between the neuroendocrine system and the immune system is depicted in Figure 1.

## 5. Intervening with the Neuroendocrine Immune System for Treatment of MS

To date, none of the available therapies for MS are curative. Their primary aims are inducing remission after relapse, reducing the number of new relapses, and preventing or slowing the progression of disability. During acute relapse, patients may be hospitalized and symptomatically treated with high doses of corticosteroids. Additionally, a number of disease-modifying treatments have been approved, albeit mostly only for RR-MS. These include IFN-*β*, glatiramer acetate, natalizumab and fingolimod. Whereas their relative success in RR-MS patients supports the role of the immune system in demyelination and axonal loss, these drugs are not sufficient to stop accumulation of disability. Management of these deficits is therefore also important [211]. Here, we will focus on treatment modalities that primarily intervene at the level of the neuroendocrine system.

### 5.1. Management of Relapse Using Glucocorticoids

Since the 1950s, GC are widely used for the suppression of inflammation in chronic inflammatory diseases such as asthma, RA, MS, and other autoimmune diseases. Despite the introduction of disease-modifying therapies, GC therapy remains the first-line treatment upon relapse for inducing remission in MS sooner and with fewer deficits for the patient. Methylprednisolone is among the most commonly used corticosteroids in MS and reduces the number of gadolinium-enhancing lesions during MS exacerbations [212]. This effect is mediated by dampening the inflammatory cascade, inhibiting the activation of T cells, and decreasing migration of immune cells into the CNS [213]. The optimal dose, frequency and duration of treatment, and route of administration of methylprednisolone are constantly being investigated for improvement of patient care. One study reported that high doses of methylprednisolone were more effective for treatment of relapses, whereas low doses of methylprednisolone correlated with disease reactivation [214]. Studies suggest that GC administered orally are equally effective at treating MS symptoms as intravenous treatment [215, 216]. To date, little is known about the effect of long-term treatment on disease progression in patients with MS [217]. Nevertheless, Then Bergh et al. have reported reduction of inflammatory disease activity and T2 lesion volume in RR-MS by a single monthly methylprednisolone infusion without clinically relevant side effects [218]. Furthermore, different combination treatment regimens are under evaluation in order to achieve synergism and improve MS management [219–221].

Although the majority of patients with MS benefits from GC treatment, a small set of patients fails to adequately respond, suggesting differences in sensitivity to GC, a phenomenon recognized as GC resistance [222]. Given the important role of endogenous GC in controlling the immune system, GC resistance may be associated with the disease course or the susceptibility of MS. However, conflicting results are reported by studies investigating *in vitro* GC resistance in MS. Whereas some have demonstrated reduced sensitivity of patients' white blood cells to GC treatment in order to suppress lymphocyte function [223], others have found no differences [224] as compared to healthy controls. Observations of reduced GC sensitivity have been made in other autoimmune diseases or inflammatory diseases, including RA and asthma [225, 226], and several factors have been identified contributing to GC resistance, such as reduced GR expression [227]. Although the mechanisms for GC resistance in MS remain to be further explored, these results may suggest implications for treatment efficacy, at least in a subgroup of MS patients.

Because of the aforementioned effects of circadian rhythms on the symptoms of autoimmune and inflammatory diseases, there is a growing interest in the efficacy of timed treatment or so-called chronotherapy. Although the impact of chronotherapeutics on treatment success remains to be fully elucidated, beneficial effects of chronotherapeutics have been identified in the management of MS and RA [228, 229] as evidenced by significantly improved clinical recovery upon nighttime treatment with GC [230].

### 5.2. Lifestyle Interventions and Physical Rehabilitation

Different lifestyle interventions can influence the neuroendocrine-immune system, including physical exercise. Physical exercise triggers a systematic series of neuroendocrine and immune events directed at accommodating the human body to the increase in physiological demands. Furthermore, the neuroendocrine-immune system can adapt to chronic overload or exercise training. Because of the vital role of the neuroendocrine system at maintaining homeostatic control during exercise, one exercise bout results in an increase of hormonal levels, including growth hormone, testosterone, cortisol, ACTH, adrenalin, noradrenalin, and estradiol [243]. On the other hand, the immune system is also important in maintaining homeostatic control during and after physical exercise. Changes that occur following an exercise bout include altered counts of peripheral blood leukocytes [244] as demonstrated by increased concentrations of neutrophils and lymphocytes [245] as well as increased serum concentrations of pro- and anti-inflammatory cytokines and acute phase proteins [246]. Furthermore, long-term exercise training has been shown to reduce basal cytokine levels and low-grade inflammation [247]. However, this could not be reproduced by others who reported no effect of long-term exercise on basal cytokine levels, albeit that a decrease of C-reactive protein (CRP) levels was observed [248].

Aforementioned observations triggered the interest to use physical exercise in MS patients in order to manage disease-related impairments. It was shown that physical exercise beneficially affects quality of life, symptoms including depression, fatigue, and possibly cognitive functions in MS patients [249]. Since it is becoming increasingly clear that these neuropsychiatric symptoms of MS are, at least in part, mediated by biological processes such as inflammation, neuroendocrine dysfunction, or regional brain damage, physical exercise may successfully affect the underlying biology and slow down the disease process [250]. Besides, several studies evaluated the effect of physical exercise on disease progression in MS patients using the expanded disability status scale (EDSS) score. In general, these studies did not found any change after either endurance training [251–253], resistance training [254–256], or combined training interventions [257, 258]. In contrast, one study reported an improvement in EDSS score upon a combined training program [259]. Alternatively, a protective effect of cardiorespiratory fitness on brain function and structure in MS patients has been demonstrated using MRI [260, 261].

To date, the mechanisms linking physical exercise and disease status in MS patients remain, however, to be elucidated [262]. It is possible that physical exercise counteracts imbalances between pro-inflammatory Th1 cytokines and anti-inflammatory Th2 cytokines [263]. A few studies have addressed the effect of physical exercise on cytokine levels in MS patients, although conflicting results were reported. On the one hand, IL-4, IL-10, CRP, and IFN-*γ* levels were reduced in MS patients after 8 weeks of biweekly resistance training [264]. Similarly, it was shown that IL-17 and IFN-*γ* levels were reduced in MS patients after 8 weeks of combined endurance and resistance training [259]. In contrast, elevated IFN-*γ* and TNF-*α* levels in MS patients after 8 weeks of endurance training were demonstrated, whereas no changes were observed in healthy controls [265]. These effects of physical training on the immune system may indirectly be mediated via modulation of the neuroendocrine system. Indeed, White et al. showed increased *β*1 and *β*2 adrenergic receptor expression in MS patients upon a moderate exercise bout as compared to controls [266].

### 5.3. Clinical Testing of New Treatment Modalities

#### 5.3.1. Estrogen

Several studies in EAE have shown the inhibitory effects of estrogens on disease pathogenesis [191, 267, 268]. Indeed, estrogen treatment before induction of EAE delays onset of disease and reduces disease activity. Protective mechanisms of estrogen treatment in EAE involve anti-inflammatory processes including decreased production of pro-inflammatory cytokines, such as TNF-*α*, and induction of Treg. Furthermore, decreased expression of MMP-9 by T cells was reported, resulting in reduced infiltration of T cells into the CNS [58]. Based on these findings, several clinical trials investigating estrogen administration in MS are underway [269, 270].

In a first pilot crossover trial, 6 female RR-MS patients were treated with 8 mg estriol per day during 6 months, followed by a 6-month posttreatment period and a subsequent retreatment period during 4 months. The investigators reported reduced number and volume of gadolinium-enhancing lesions upon estriol treatment [269]. A multicenter randomized double-blind placebo-controlled phase II trial was recently started at the University of California in order to investigate the therapeutic effect of oral estriol treatment in combination with glatiramer acetate treatment in female RR-MS patients (http://www.clinicaltrials.gov/ct2/show/NCT00451204). The European POPART'MUS study, an ongoing double-blind placebo-controlled phase III trial, designed for women with MS in their postpartum period, aims at the reduction of postpartum relapses by administration of estradiol and progestin. High doses of progestin in combination with endometrial-protective doses of estradiol will be given immediately after delivery and continuously during the first three months postpartum [270]. Although the first results of therapeutic use of estrogen in MS are encouraging, more research is warranted in order to understand the estrogen-mediated underlying mechanisms. The outcomes of the currently ongoing MS trials may help to clarify therapeutic use of estrogen in combination with first-line immunomodulatory drugs.

For completeness, also the effect of testosterone was evaluated in a first pilot study including 10 men with RR-MS. A daily treatment with 10 g of a 100 mg testosterone-containing gel for 12 months resulted in improvement of cognitive performance and delayed progression of brain atrophy. These findings suggest that testosterone treatment is safe and well-tolerated and may have neuroprotective effects in men with RR-MS [271].

#### 5.3.2. Neurotransmitters

(1) *Catecholamines.* By increasing noradrenalin levels through administration of tri- and tetracyclic antidepressants and L-dopa, the course of MS was ameliorated [272]. Indeed, after 1-2 months of treatment approximately 75% of patients experienced substantial improvements in sensory, motor, and autonomic symptoms. Moreover, these patients regained functions that were lost for several years. Interestingly, also treatment with IFN-*β*, which is a widely used and approved immunomodulatory therapy for MS, was shown to substantially elevate the catecholamine levels in PBMC of MS patients [273]. This suggests that the improvement in MS during IFN-*β* treatment is, at least in part, mediated by increased levels of catecholamines.

(2) *Acetylcholine.* Based on experimental evidence that ACh promotes production of anti-inflammatory cytokines [109], it was demonstrated that a cholinesterase inhibitor can alleviate neuroinflammatory responses in the EAE model thereby reducing clinical and pathologic severity of EAE [274]. In several phase I/II clinical studies using cholinesterase inhibitor therapy, beneficial effects on cognitive deficits in MS were observed [275, 276]. Indeed, following treatment with rivastigmine, a widely used ACh esterase inhibitor for the treatment of Alzheimer's disease, Shaygannejad et al. reported a modest, but significant improvement of memory in MS patients with Wechsler Memory Scales (WMS) confirmed mild verbal memory impairment [276]. Nevertheless, similar improvements were observed in placebo-treated MS patients. Additionally, treatment of MS patients with donepezil, an alternative ACh esterase inhibitor, showed significant improvement in memory performance on the selective reminding test, a test of verbal learning and memory, as compared to placebo-treated MS patients. Moreover, cognitive improvement was reported by clinicians in twice as many donepezil versus placebo-treated MS patients. In addition, the donepezil-treated MS patients themselves reported more often memory improvement than placebo-treated MS patients [275].

(3) *Glutamate.* Since extracellular accumulation of glutamate contributes to excitotoxic injury of neurons and glial cells, inhibition of glutamate might be beneficial in MS patients. For this, Killestein et al. examined the effect of one year riluzole treatment in MS patients [277]. Riluzole is a neuroprotective agent that inhibits the release of glutamate from nerve terminals. Moreover, it modulates iGluR and inhibits excitotoxic injury in several experimental models of neurodegenerative disease [278]. The investigators reported a reduction in the rate of brain and cervical cord atrophy as well as in the development of T1 hypointense lesions on MRI in primary progressive MS.

(4) *Serotonin.* Experimental evidence from animal studies has shown an immunosuppressive role of serotonin in autoimmunity. Sijens et al. evaluated the impact of elevated extracellular levels of serotonin mediated by fluoxetine, a selective serotonin reuptake inhibitor used as antidepressant, in MS patients [279]. By using diffusion tensor imaging (DTI) and ^1^H magnetic resonance spectroscopy (MRS), the investigators reported partial normalization in diffusion and metabolic properties of brain tissue upon 2-week treatment with fluoxetine, thereby providing evidence for a possible neuroprotective effect of fluoxetine in MS.

(5) *Histamine.* Ligation of the histamine receptor, H1R, on immune cells induces secretion of pro-inflammatory cytokines, such as IL-1*β*, IL-6, IL-8, and the chemokine CCL5 [152]. Therefore, treatment with H1R antagonists would reduce the secretion of pro-inflammatory cytokines. Indeed, treatment of MS patients with hydroxyzine, a well-known H1R antagonist, stabilized or improved the neurological status of 75% of treated MS patients, as assessed by Kurtzkes's EDSS [280].

#### 5.3.3. Cytokines

In 1993, interferon (IFN)-*β* was the first product to be approved by the FDA as disease-modifying treatment for MS. To date, these include three different commercial formulations which have been demonstrated to reduce the inflammatory process in MS by decreasing the secretion of pro-inflammatory cytokines, increasing anti-inflammatory cytokine levels, and reducing the number of immune cells migrating towards the CNS. In doing so, IFN-*β* decreases relapse rate, increases time between relapses, and decreases the severity of relapses, while decreasing the amount of accumulated lesions seen on MRI.

In addition, targeting cytokine production has been intensively investigated as a potential treatment strategy in autoimmunity [281]. One of the greatest successes in immunology is the treatment of RA with anti-TNF-*α* therapy. Unfortunately, TNF neutralization in MS patients exacerbated disease symptoms [282]. Similarly, treatment with tocilizumab and anakinra, humanized monoclonal antibodies competing for receptor binding with IL-6 and IL-1*β*, respectively, has been approved in RA. However, the safety and efficacy of anakinra, tocilizumab, or administration of other IL-1- and IL-6-targeting compounds have not yet been evaluated in MS patients [283]. Furthermore, also IL-12 and IL-23, interleukins sharing p40 as a common subunit, have a clear role in the pathogenesis of MS because of their respective function in Th1 and Th17 differentiation. Ustekinumab, a monoclonal antibody that neutralizes the p40 subunit, is effective in patients with psoriasis or psoriatic arthritis, and in patients with Crohn's disease. Unfortunately, ustekinumab failed to show any efficacy in RR-MS patients [284]. In summary, although targeting cytokines as therapy for MS is a feasible approach, careful consideration must be given to the highly pleiotrophic character of the cytokine as well as the stage of the disease process being targeted.

## 6. Conclusion

Although knowledge of the immunopathogenesis as well as genetic predisposition of MS has greatly increased over the last decades, potential environmental triggers such as stress and pregnancy may not be underestimated in order to better understand how these factors modulate disease. In this perspective, it is clear that the neuroendocrine-immune system has an important role in the pathogenesis of autoimmune diseases, including MS. Here we have provided an overview of the complex system of crosstalk between the neuroendocrine and immune system, whereby they share an extensive range of common messenger molecules and receptors and whereby they can monitor each other's activities. Discrepancies at any level can lead to changes in susceptibility to and to severity of several autoimmune and inflammatory diseases. These principles are now being used to test novel therapies for MS based on addressing and correcting the dysregulation of these neural and neuroendocrine pathways.

However, the key question that remains unanswered is whether these alterations in neuroendocrine pathways and receptors are involved in the pathogenesis of MS as a predisposing factor or whether they are a result of the inflammatory status of the disease. Based on preliminary evidence that hormonal changes may appear before the symptomatic phase of the disease [285, 286], it is tempting to speculate that a pro-inflammatory hormone favors the rupture of tolerance, which is a key feature of autoimmunity.

In conclusion, dysfunction of the neuroendocrine-immune system in patients with autoimmune diseases, including MS, seems to be important in the pathogenesis of these diseases. Increasing the knowledge of the neuroendocrine-immune system in MS can help to elucidate the underlying mechanisms of the inflammatory responses in MS and mutatis mutandis in other autoimmune diseases. Furthermore, intensive research on the modulatory function of the neuroendocrine-immune system may provide new therapeutic approaches for the treatment of MS in the near future.

## Figures and Tables

**Table 1:** Neuroendocrine factors and their immunomodulatory effects.

Substance	Receptor	Effect on immune response	Reference
Acetylcholine	Muscarinic acetylcholine receptor (mAChR)	Differentiating towards a Th1 phenotype	[111]
	Nicotinic acetylcholine receptor (nAChR)	Inhibits IL-1*β*, IL-6, IL-18, and TNF-*α* production	[109]

ACTH	ACTH receptor	Inhibits IFN-*γ* production and Ig production and blocks macrophage activation by IFN-*γ*	[231]

Adrenalin/ noradrenalin	*α*-Adrenergic receptors	Upregulation of cAMP; inhibits IL-1, IL-6, IL-12, and TNF-*α* production; enhances IL-10 production	[91]
	*β*-Adrenergic receptors	Downregulation of cAMP	[93, 94]

Cortisol	Glucocorticoid receptor (GR)	Inhibits IFN-*γ*, IL-2, IL-6, and TNF-*α* Enhances IL-4 and TGF-*β* production Enhances immune cell expression of IL-1, IL-2, IL-6, and IFN-*γ* receptors	[10, 232, 233]

CRH	Corticotropin-releasing hormone receptor	Activates macrophages Inhibits IL-1 and IL-6 production	[231]

Dopamine	D1-like receptors	Upregulation of cAMP	[99]
	D2-like receptors	Downregulation of cAMP	[99]

GABA	GABA receptors	Reduces the proliferative response of activated CD8^+^ T cells Reduces IL-6 release	[169, 170]

Glutamate	mGluR1	Enhances IL-2, IL-6, IL-10, TNF-*α*, and IFN-*γ* production	[116]
	mGluR5	Inhibits IL-6 production	[116]

Growth hormone	Growth hormone receptor	Activates macrophages and enhances H_2_O_2_ production	[234]

Gonadotropin-releasing hormone	Gonadotropin-releasing hormone receptor	Increases IL2R expression, T- and B-cell proliferation, and serum Ig	[235, 236]

Histamine	Histamine 1 receptor, histamine 4 receptor	Enhances IL-1*β*, IL-6, IL-8, and RANTES production Induce chemotaxis of CD4^+^ T cells	[152–154]
	Histamine 2 receptor	Inhibits IL-12, IFN-*γ* and TNF-*α*, and enhances IL-10 production	[155]

Luteinizing hormone	Luteinizing hormone/choriogonadotropin receptor	Enhances IL-2 stimulated T-cell proliferation	[237]

Melatonin	Melatonin receptor	Enhances IL-1, IL-2, IL-6, and IFN-*γ* production	[238, 239]

Neurokinin A	Neurokinin 2 receptor (NK2-receptor)	Enhances mRNA expression of IFN-*α* and IFN-*β* Enhances DC function	[138]

Estrogen	Estrogen receptor	Enhances T-cell proliferation and activity IFN-*γ* gene promotor	[240]

Progesterone	Progesterone receptor	Enhances IL-4 production and CD30 expression	[85, 86]

Prolactin	Prolactin receptor	Enhances T cell proliferation, IFN-*γ*, IL-2 receptor expression, and macrophage function	[42, 43]

Serotonin	Serotonin-1a receptor	Enhances NK cell cytotoxicity Downregulation of cAMP Stimulation of T-cell proliferation	[142, 143]
	Serotonin-2a receptor	Inhibits lymphocyte proliferation	[144]

Substance P	Neurokinin 1 receptor (NK1-receptor)	Enhances IL-1*β*, IL-6, TNF-*α*, MIP-1*β*, and IFN-*γ* production Enhances T-cell proliferation Enhances NK cell cytotoxicity	[129, 130, 132]

Vasopressin	Vasopressin receptor	Enhances IFN-*γ* production	[241]

VIP	Vasoactive intestinal peptide receptor	Inhibits T-cell proliferation and IL-12 Enhances IL-5 and cAMP production	[242]
